# 2325. Five-day quarantine of patients exposed to SARS-CoV-2 within hospitals in the Omicron variant era

**DOI:** 10.1093/ofid/ofad500.1947

**Published:** 2023-11-27

**Authors:** Eun Ju Choo

**Affiliations:** Soonchunhyang University Bucheon Hospital, Bucheon, Kyonggi-do, Republic of Korea

## Abstract

**Background:**

Since severe acute respiratory syndrome coronavirus 2 (SARS-CoV-2) can be transmitted even in the absence of symptoms, it can be difficult to prevent its transmission within healthcare facilities. This study aimed to investigate whether initial screening tests and quarantine are still necessary for in-hospital close contact patients in the Omicron-dominant era.

**Methods:**

This retrospective, observational study was performed at a tertiary hospital in South Korea, comprising mostly multi-bed rooms, with four or five beds each. Real-time reverse transcriptase polymerase chain reaction (RT-PCR) for SARS-CoV-2 was performed on the first and fourth day of quarantine, using a PowerChekTM 2019-nCoV Real-time PCR Kit (Kogenebiotech, Seoul, Korea) or an Allplex 2019-nCoV Assay (Seegene, Seoul, Korea). If relevant symptoms developed during quarantine, testing was promptly performed, regardless of the established schedule.

**Results:**

Between June 15, 2022 and December 29, 2022, there were 179 events of COVID-19 within the hospital. Eighteen exposed patients were excluded because they were discharged during quarantine, and 705 patients were included in the final analysis. The median number of exposed inpatients was 4 (interquartile range, 2 – 5) for each event. Fifty-six (7.9%; 95% confidence interval [95%CI], 6.0 – 10.3%) exposed patients had positive initial screening tests. Among those with an initially negative test, positive conversion was identified in 31 patients (4.8%; 95%CI, 3.2 – 6.8%) during quarantine. In correlation with the number of confirmed COVID-19 cases in South Korea at that time, the number of in-hospital events and exposed patients peaked in August 2022. However, positive test rates at initial testing or during quarantine did not differ significantly according to the month (*P* = 0.095 and *P* = 0.104, respectively). This study showed that 12.3% of exposed patients had a positive PCR test, either at initial screening or during quarantine.

**Figure:**
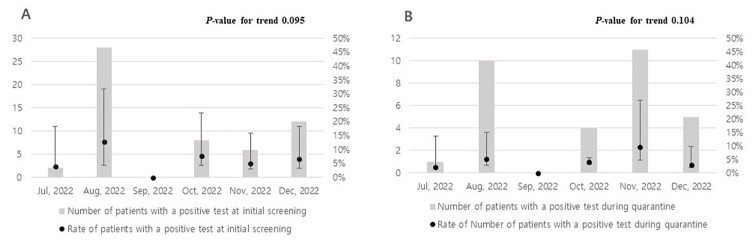

**Conclusion:**

This study suggests that screening and quarantine are still necessary for close contact inpatients in the Omicron variant era. Testing intervals and the duration of quarantine may need adjustment considering the current shortage of isolation rooms, testing capacity, and healthcare personnel.

**Disclosures:**

**All Authors**: No reported disclosures

